# Differences in the preferential location and invasiveness of diffuse low‐grade gliomas and their impact on outcome

**DOI:** 10.1002/cam4.3216

**Published:** 2020-06-14

**Authors:** Francesco Latini, Markus Fahlström, Göran Hesselager, Maria Zetterling, Mats Ryttlefors

**Affiliations:** ^1^ Department of Neuroscience, Neurosurgery Uppsala University Uppsala Sweden; ^2^ Department of Surgical Sciences, Radiology Uppsala University Uppsala Sweden

**Keywords:** astrocytomas, Brain‐Grid, cerebral gliomas, oligodendrogliomas, white matter

## Abstract

**Background:**

Low‐grade gliomas (LGGs) are primary diffuse slow‐growing brain tumors derived from glial cells. The management of these tumors is dependent on their location, which often harbors eloquent areas. We retrospectively recorded the location of diffuse gliomas to identify whether specific differences exist between the histological types.

**Methods:**

We analyzed 102 patients with previous histological diagnosis of WHO‐II astrocytomas (62) and WHO‐II oligodendrogliomas (40) according to WHO‐2016 classification. MRI sequences (T2‐FLAIR) were used for tumor volume segmentation and to create a frequency map of their locations within the Montreal Neurological Institute (MNI) space. The Brain‐Grid (BG) system (standardized radiological tool of intersected lines according to anatomical landmarks) was created and merged with a tractography atlas for infiltration analysis.

**Results:**

Astrocytomas frequently infiltrated association and projection white matter pathways within fronto‐temporo‐insular regions on the left side. Oligodendrogliomas infiltrated larger white matter networks (association‐commissural‐projection) of the frontal lobe bilaterally. A critical number of infiltrated BG voxels (7 for astrocytomas, 10 for oligodendrogliomas) significantly predicted shorter overall survival (OS) in both groups. Bilateral tumor extension in astrocytomas and preoperative tumor volume in oligodendrogliomas were independent prognostic factors for shorter OS.

**Conclusions:**

Astrocytomas and oligodendrogliomas differ in preferential location, and this has an impact on the type and the extent of white matter involvement. The number of BG voxels infiltrated reflected different tumor invasiveness and its impact on OS in both groups. All this new information may be valuable in neurosurgical oncology to classify and plan treatment for patients with diffuse gliomas.

## Graphical abstract

Astrocytomas preferentially infiltrate white matter of the fronto‐temporo‐insular region. Oligodendrogliomas tend to invade fibers in fronto‐mesial regions bilaterally. Tumor invasiveness as expressed by the number of invaded BG has an impact on OS.

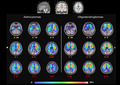

## 1 INTRODUCTION

Central nervous system (CNS) tumors are defined by their cell of origin and their histopathological characteristics, which predict their behavior.^1^ Low‐grade gliomas (LGGs) are WHO grade II tumors that affect mostly adult patients and include diffuse astrocytomas and diffuse oligodendrogliomas.^2^, ^3^ The clinical course of low‐grade gliomas is diverse with a peak incidence around 30‐40 years and the tendency to recur or to transform into high‐grade gliomas.^4^

The management of these tumors is dependent on their location. Several studies have described LGGs as being located preferentially in “secondary” functional areas (immediately near the so‐called primary eloquent regions), especially within the SMA (supplementary motor area) and the insular lobe. This preferential localization has been explained as being due to developmental, cyto‐ and myeloarchitectonic, neurochemical, metabolic, and functional reasons.^5^, ^6^, ^7^ However, many of the studies investigating the radiological location of gliomas included different grades and/or different histological subtypes, without any evidence suggesting specific differences between them.^5^, ^6^, ^7^ The authors of other studies have suggested differences in the molecular biology of LGGs as the reason for differences in tumor location,^8^, ^9^, ^10^ with a higher rate of 1p deletion in the anterior part of the brain (in particular in the frontal lobe)^9^ and a lower rate in the insula,^8^ or the absence of IDH1 mutation within the insula^10^ and its presence in tumors located within the frontal lobe.^11^ At the biological level, these results support the existence of differences in cortical‐subcortical infiltration and/or dislocation patterns in relation to the histological origin that may depend on permissive neighboring structures around the tumor. Dissemination of astrocytic tumors seems to be confined to white matter by the cortex or deep gray nuclei, which, under certain circumstances, act as barriers to invasion of some gliomas.^12^, ^13^ This phenomenon is less prominent in oligodendrogliomas, which will frequently invade the cortex and are less likely to respect anatomic boundaries.^14^ The grade of the tumor, however, does not seem to be strictly correlated with the degree of local invasion, and low‐grade astrocytomas (WHO II) may show extensive infiltration of adjacent subcortical regions.^15^ The implications with regard to the exact location, invasive behavior, and histological correlation are particularly important for treatment planning and the outcome.

The aim of this study was to retrospectively record the exact location of histologically diagnosed WHO II astrocytomas and WHO‐II oligodendrogliomas and identify whether specific radiological or topographical differences between the histological types could affect the outcome. The white matter architecture within the preferential locations was analyzed to describe whether specific functional networks were preferentially invaded by the tumor subgroups.

## 2 MATERIALS AND METHODS

### 2.1 Patient population

We collected clinical and radiological data for all patients who had undergone surgery between February 2005 and December 2015. One hundred and two adult patients (>18 years) with a radiological and histological diagnosis of low‐grade glioma (WHO II), were included in this study and reclassified according to the WHO 2016 classification.^1^ Molecular verification including the IDH status (either IDH1/IDH2 mutant, IDHm; IDH wildtype, IDHwt) and LOH1p19q codeletion were recollected in all the available cases. The regional ethics committee, approved the study protocol.

### 2.2 Clinical and radiological data

Magnetic resonance imaging (MRI) was performed on a 1.5‐T or 3.0‐T scanner, including high‐resolution T1‐weighted (3D T1 image), T2‐weighted fluid attenuated inversion recovery (T2 FLAIR) acquired in 2D or 3D, and T2‐weighted turbo spin echo (T2 TSE) and either FLAIR or 3D FLAIR images. Slice thickness ranged between 1 and 5 mm, and the axial resolution was 0.5‐2 mm. Morphological MRI findings presenting with high signal intensity on T2 FLAIR and 3D T1 images with no or minimal (patchy and faint) contrast enhancement suggestive of a low‐grade glioma were analyzed. All MR images were reevaluated by the first author of this study and reclassified according to the Brain‐Grid system.^16^

T2 TSE or 2D/3D T2 FLAIR images (preferably 3D‐based when available) in Vue picture archiving and communication system (PACS) software (version 11.1.0, Carestream Health Inc) were used for volume calculation of the lesions with the aid of a semiautomatic method (Livewire Algorithm).^16^, ^17^ The software is supported by an algorithm that uses an active contour model in order to evolve and segment the lesions. In defining the volume of the surface voxels, a clear difference in pixel contrast (black/white) assisted the operator, increasing the ability to better adapt or correct the ischemic contour line even where it was less defined. The tumors were considered bulky, with well‐defined margins (without any finger‐like hyperintense signals on T2 TSE or T2 FLAIR sequences), while tumor margins with unclear and irregular signal intensity on T2 FLAIR sequences were considered diffuse.

Furthermore, T2 TSE or T2 FLAIR images of all the patients were normalized to Montreal Neurological Institute (MNI) space using the built‐in normalizing software in DSI studio (DSI Studio, http://dsi‐studio.labsolver.org/download‐images).

### 2.3 Probabilistic and frequency maps

The total lesion was successively manually segmented on original FLAIR images by the first author with DSI studio within the MNI space.^18^ Statistical maps of tumor location frequency were obtained by computing the cumulative number of observed lesions for each voxel and divide by the total amount of lesions.^19^

### 2.4 Brain‐Grid analysis

**Figure 1:**
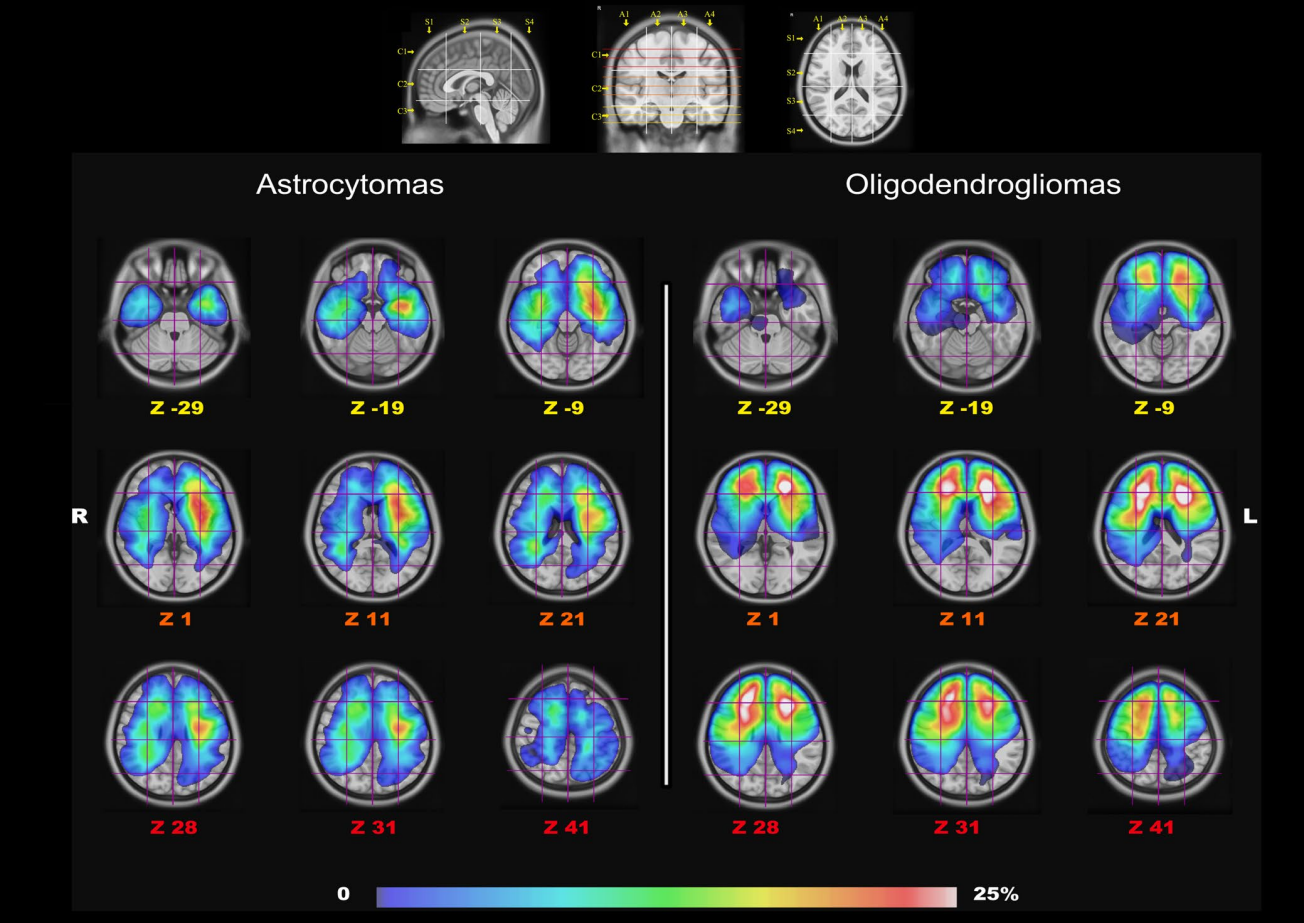
The image shows the gradient maps reconstructed from the fusion of each tumor region within the MNI space (*Z* coordinates for each slice). In the upper part, the Brain‐Grid System used as a reference for BG voxels count with sagittal, coronal and axial projection of the BG lines. In the lower part, the frequency of tumor location for the two populations is color graded (0%‐25% in the gradient scale) according to the rate of voxel infiltration

The Brain‐Grid was created intersecting longitudinal lines on the axial, sagittal, and coronal planes on a T1‐weighted average brain in the MNI space.^16^ Superficial and consistent cortical/gyral anatomical landmarks were chosen based on their bilateral symmetry and their relationship with the subcortical white matter architecture. The sulcus between the cingulum cortex and corpus callosum, the mammillary bodies, superior temporal sulcus, middle frontal sulcus, the midline, the parieto‐occipital sulcus, the temporal‐occipital junction, the anterior and posterior insular point were selected as clear and consistent landmarks. The Brain‐Grid was constructed by three axial lines, two coronal lines, and three sagittal lines, whereas the intersection of these lines creates 48 grid voxels. Each voxel could be identified using simple nomenclature with radiological orientation. In the axial (A) plane, voxels are labeled 1‐4, right to left direction. In the coronal (C) plane, voxels are labeled 1‐3, cranio‐caudal direction. In the sagittal (S), voxels are labeled 1‐4, anterior‐posterior direction.^16^ In this study, the Brain‐Grid analysis was performed in the MNI space using the built‐in software of DSI studio (DSI Studio, http://dsi‐studio.labsolver.org/download‐images). The number of BG voxels involved by tumor lesion was recorded applying the Brain‐Grid classification system in the MNI space using DSI studio (Figure 1). The tumor lesions were reclassified according to the Brain‐Grid system to obtain quantitative and qualitative data.

### 2.5 HCP 1021

The WU‐Minn HCP consortium is an institutional, review board‐approved, NIH‐funded project.^20^ A group average template was constructed from a total of 1021 subjects. A multishell diffusion scheme was used. The *b*‐values were 990, 1985, and 2980 s/mm^2^ and the number of diffusion sampling directions were 90, 90, and 90, respectively. The in‐plane resolution and the slice thickness were 1.25 mm. The diffusion data were reconstructed in the MNI space using q‐space diffeomorphic reconstruction^21^ to obtain the spin distribution function.^22^ A diffusion sampling length ratio of 2.5 was used, and the output resolution was 1 mm. The restricted diffusion was quantified using restricted diffusion imaging.^23^

Major projection, commissural, and association white matter pathways were reconstructed within the HCP 1021 template following the anatomical criteria previously published with the Brain‐Grid DTT reference atlas^16^ and matched with gradient maps for tumor frequency.

### 2.6 Statistical analysis

Frequency distributions and summary statistics were calculated for all variables. The variables analyzed were age, side of tumor location, description of radiological border (bulky or diffuse), preoperative volume, number of BG voxels, invasion of eloquent white matter pathways,^19^ and extent of resection (EOR). To investigated possible differences between subgroups of astrocytomas and oligodendrogliomas according to their molecular status against the not confirmed groups (NOS, non‐otherwise specified), we used Kruskal‐Wallis test between IDHm, IDHwt, and astrocytomas NOS for numerical variables. Mann‐Whitney *U* test for independent samples was used for comparison between confirmed oligodendrogliomas (LOH1p19q+) and oligodendrogliomas NOS for numerical variables. Pearson's chi‐squared test was used for categorical variables analysis between subgroups.

To investigate differences in variables distribution between astrocytomas and oligodendrogliomas, a Mann‐Whitney *U* test for independent samples was used for group comparison for numerical variables. Pearson's chi‐squared test was used for categorical variables.

We calculated survival probability using the Kaplan‐Meier method,^24^ performing comparisons with the log‐rank test to assess the effect of variables on overall survival (OS). OS was calculated from dates of biopsy or surgical intervention to death or last follow‐up. For the number of BG voxels and tumor volume, an optimal cutoff choice in two groups was made according to receiver operating characteristics curves (ROC).^25^ We examined all variables in the proportional hazard analysis (Cox model^26^), to identify independent predictors of survival.

Univariate OS analyses using proportional hazards models were used to assess the prognostic significance of multiple variables. Forward step‐wise proportional hazards modeling was performed to assess the relative and independent prognostic capacity of each parameter. A second block was used for interaction analysis between the more relevant categorical variables (infiltration of crucial white matter, side, radiological border, IDH status in astrocytomas and LOH status in oligodendrogliomas) and numerical variables (volume, number of BG voxels, EOR). All statistical analyses were performed at a significance level of *P* < .05. For analyses involving multiple categories, to minimize the problem of overstatement of statistical differences resulting from multiple comparisons, individual comparisons were considered for statistical significance only if the overall test was statistically significant. The statistical analysis was performed using the statistical package SPSS 25.0 (SPSS, Inc).

## 3 RESULTS

### 3.1 Patients and tumor characteristics

A total of 102 patients were retrospectively analyzed; according to the histology, 62 were diagnosed with astrocytomas WHO grade II and 40 with oligodendrogliomas WHO grade II. A molecular analysis confirming the histological diagnosis according to WHO‐2016 classification^1^ was performed in all the previously classified oligo‐astrocytomas WHO‐II (14 cases of which 6 were oligodendrogliomas and 8 were astrocytomas). In 41/62 astrocytomas, the molecular analysis was available revealing 25 IDHm profile and 16 IDHwt. In the remaining 21 cases the IDH status was unavailable and therefore classified as astrocytomas NOS. In 26/40 oligodendrogliomas, the diagnosis was confirmed by molecular analysis (IDH mutation and LOH1p19q+). The patients without molecular confirmation were, however, included into the histological subgroups (matching radiological criteria, histological criteria, and proliferation index level <5%) but considered as diffuse astrocytomas NOS and diffuse oligodendrogliomas NOS.^1^ Clinical and radiological characteristics are presented in Table 1. The mean follow‐up was 6.4 years (range 0.5‐17 years).

**Table 1:** Summary of the clinical and radiological data in the two populations. Independent samples Mann‐Whitney *U* test was used for numerical variables for the two populations

Clinical and radiological variables	Astrocytomas	Oligodendrogliomas	*P‐*value
Number of patients	62	40	
Mol‐confirmed/NOS (IDHm‐IDHwt)	41/21 (25‐16)	26/14	
Age (y)			.231
Median (IQR) (range)	41.5 (28‐56) (19‐75)	43 (33‐53) (23‐65)	
Gender			
Male ‐ n (%)	35 (56)	25 (62)	
Female ‐ n (%)	27 (44)	15 (38)	
Epilepsy			
Yes ‐ n (%)	51 (82)	28 (70)	
No ‐ n (%)	11 (18)	12 (30)	
Surgical indication		30 (75)	
Resection ‐ n (%)	41 (66)	10 (25)	
Biopsy ‐ n (%)	21 (34)		
Surgical resection %			
Median (IQR)	78 (62‐98)	86 (69‐100)	.231
Survival from diagnosis years			.583
Median (IQR)	6 (4‐8.2)	6 (4‐8)	
Volume mL			
Median (IQR)	47 (20‐108)	62 (34‐112)	.103
Brain‐Grid voxels n	7 (4‐10)	8 (6‐10.7)	.128
Median (IQR)			

			Chi‐squared	*df*	
Radiological border					
Bulky ‐ n (%)	24 (38.7)	11 (27.5)			
Diffuse ‐ n (%)	38 (61.3)	29 (72.5)	1.355	1	.244
Infiltrated eloquent WM^a^					
Infiltrated ‐ n (%)	18 (29)	15 (37.5)	0.797	1	.372
Not infiltrated ‐ n (%)	44 (71)	25 (62.5)			
Side					
Left ‐ n (%)	33 (53.2)	16 (40)			
Right ‐ n (%)	24 (38.7)	20 (50)	0.162	2	.922
Bilateral ‐ n (%)	5 (8.1)	4 (10)			

Note

Categorical variables were analyzed with Pearson's chi‐squared test.

Abbreviations: %, percentage within the same variable group; BG, Brain‐Grid system; *df*, degree of freedom; F, female subjects; IDHm, IDH mutant; IDHwt, IDH wild type; IQR, interquartile range; M, male subjects; Mol‐confirmed, histological diagnosis confirmed by additional molecular analysis (IDH—mutation for Astrocytomas and codeletion 1p19q for Oligodendrogliomas); n, number; WM, white matter.

^a^ Eloquent white matter refers to the study on intraoperative cortical‐subcortical mapping illustrating the “minimal common brain”.^19^

* Significance level of <.05 and confidence interval of 95%.

### 3.2 Astrocytomas and oligodendrogliomas subgroup analysis

No significative differences were detected in astrocytomas group among IDHm, IDHwt, and NOS regarding age (*P* = .284), survival from diagnosis (*P* = .292), number of BG voxels (*P* = .123), volume (*P* = .504), EOR (*P* = .622), radiological borders (*P* = .089), and eloquent white matter infiltration (*P* = .58). In the oligodendrogliomas' group, no difference was displayed between LOH1p19q+ and NOS for age (*P* = .190), survival from diagnosis (*P* = .878), number of BG voxels (*P* = .474), volume (*P* = .492), EOR (*P* = .156), radiological borders (*P* = .399), and eloquent white matter infiltration (*P* = .866). To test possible differences in outcome between subgroups a log‐rank Mantel cox analysis was performed. In neither astrocytomas (*χ* ^2^ = 0.584; *P* = .747) nor oligodendrogliomas subgroups (*χ* ^2^ = 0.16; *P* = .9) a significative difference was found.

### 3.3 Radiological features and Brain‐Grid analysis

According to the similar distribution of variables between the molecular confirmed and NOS subgroups, the radiological/topographical analysis was further conducted on two main groups based on their histological profile. No significant difference between astrocytomas and oligodendrogliomas with regard to the frequency of radiological and topographical features was detected. Results are summarized in Table 1. The Brain‐Grid classification system was applied in all MRI scans. The quantitative analysis showed a median of 7 BG voxels infiltrated for the astrocytomas and 8 for the oligodendrogliomas. The qualitative analysis demonstrated that the A3‐4C1S2 voxels (frontal cortical‐subcortical SMA on the left side) and the A3‐4C2‐3 S2 (cortical‐subcortical insular region on the left side) had the highest rate of invasion in patients with diffuse astrocytomas. In patients with oligodendrogliomas, the A2‐3C2‐S1‐2 (fronto‐mesial/fronto‐striatal regions on both sides) were the most invaded BG voxels. The ROC curves for radiological variables applied to survival, displayed an optimal cutoff value for tumor volume at 47 mL for astrocytomas and 62 mL for oligodendrogliomas, while the number of BG voxels optimal for a cutoff analysis was 7 BG voxels for astrocytomas and 10 for oligodendrogliomas.

### 3.4 Gradient maps

**Figure 2:**
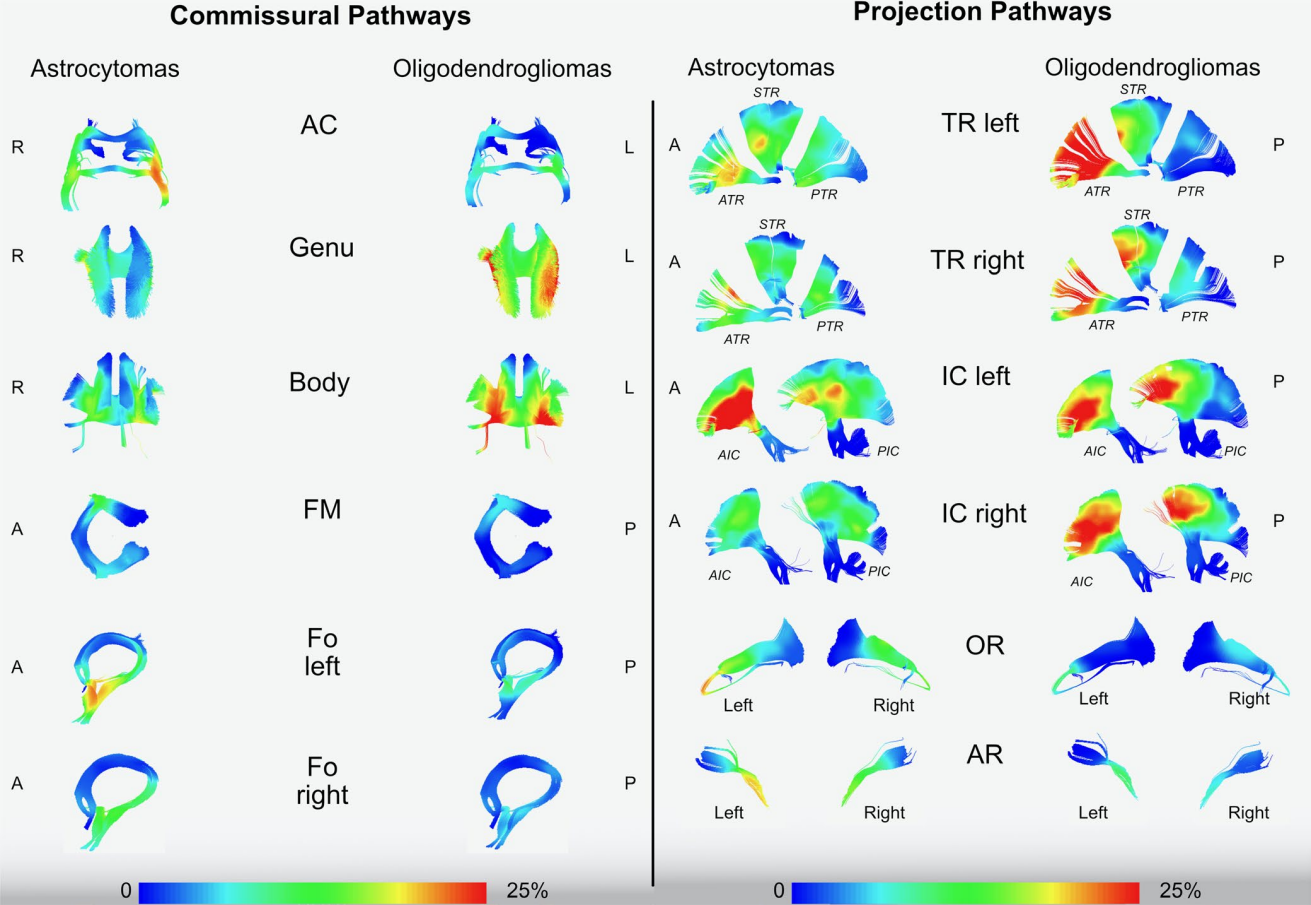
The image shows the frequency of invasion of commissural and projection pathways in astrocytomas and oligodendrogliomas using a gradient scale from the voxel‐based analysis (0%‐25% in the gradient scale) according to the rate of voxel infiltration. All the white matter pathways are tracked in MNI space according to the Brain‐Grid tractography Atlas.^16^ AC, anterior commissure; Genu, anterior portion of corpus callosum; Body, middle portion of corpus callosum; FM, forceps major (posterior part of corpus callosum); Fo, fornix; R, right; L, left; A, anterior; P, posterior; TR, thalamic radiation; IC, fibers crossing the internal capsule; OR, Optic radiation; AR, Acoustic radiation

**Figure 3:**
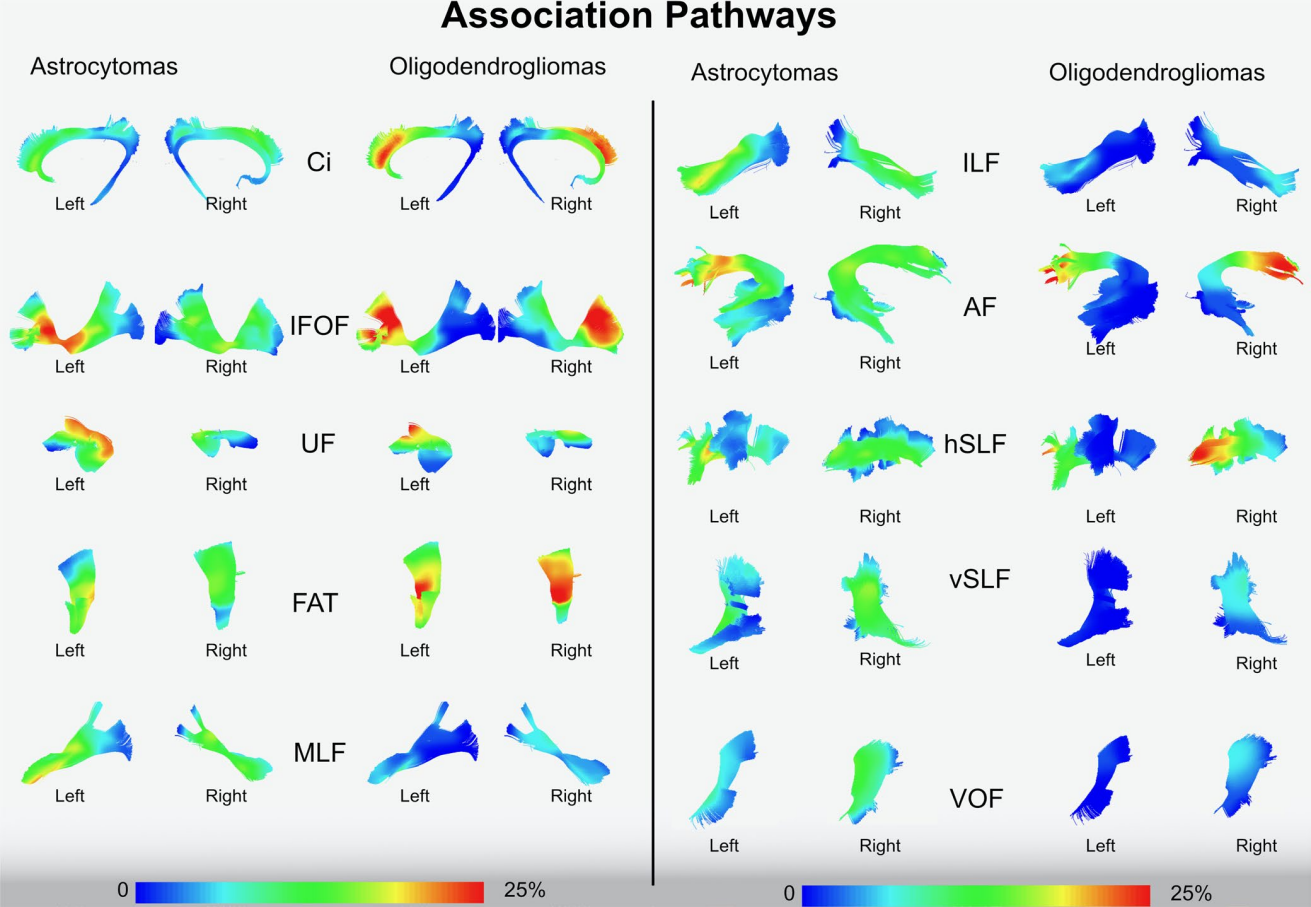
The image shows the frequency of invasion of association pathways in astrocytomas and oligodendrogliomas using a gradient scale from the voxel‐based analysis (0%‐25% in the gradient scale) according to the rate of voxel infiltration. All the white matter pathways are tracked in the MNI space according to the Brain‐Grid tractography Atlas.^16^ Ci, Cingulum; IFOF, Inferior Fronto‐occipital fasciculus; FAT, Frontal Aslant tract; MLF, Middle longitudinal fasciculus; ILF, inferior longitudinal fasciculus; AF, Arcuate fasciculus; hSLF, horizontal indirect component of superior longitudinal fasciculus; vSLF, vertical indirect component of superior longitudinal fasciculus; VOF, vertical occipital fasciculus

Two gradient maps were built within the MNI space, merging all the tumor ROIs for astrocytomas and oligodendrogliomas (Figure 1). The highest tumor index was identified for astrocytomas within the fronto‐temporo‐insular region on the left side. Within the frontal and temporal regions, the gradient was higher at the subcortical level, whereas the insula was involved both cortical and subcortical. For oligodendrogliomas, the gradient maps displayed a high tumor index in both the frontal lobes but more specifically within the deep white matter of both sides, with symmetrical distribution. A qualitative analysis of the white matter tracts within the most invaded voxels was also performed with a gradient index for tumor frequency according to each white matter fiber system (Figures 2 and 3).

### 3.5 Prognostic factors correlated with OS

**Figure 4:**
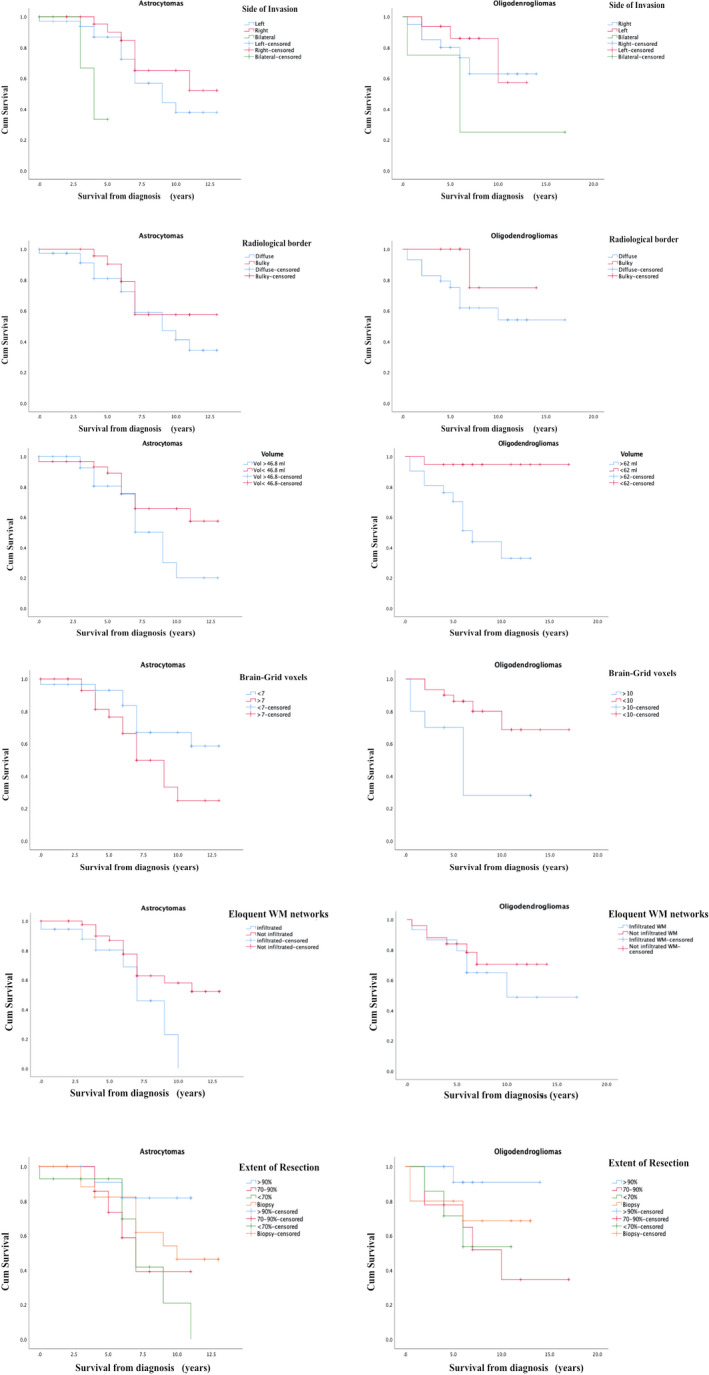
The image shows the result of survival probability using the Kaplan‐Meier method,^24^ performing comparisons with the log‐rank test to assess the effect of variables on overall survival (OS). The Kaplan‐Meier graphs show the difference in OS in relation to side of invasion, radiological border, tumor volume cutoff, Brain‐Grid voxels cutoff, infiltration of eloquent white matter, and extent of resection. See text for details

The median survival in both astrocytomas and oligodendrogliomas was 6 years (interquartile range (IQR) 4‐8.2 years for astrocytomas and 4‐8 years for patients with oligodendrogliomas). OS at 5 years was 85% for the astrocytoma group and 82% for the oligodendroglioma group. Overall survival at 10 years was 47% for the astrocytoma group and 60% for the oligodendroglioma group. In the univariate analysis, we used the Kaplan‐Meier method, with comparisons using the log‐rank test (Figure 4) and side of invasion, preoperative tumor volume, number of BG voxels, infiltration of crucial white matter networks, EOR as explanatory variables.

In patients with astrocytomas, a shorter OS was demonstrated if bilateral invasion was detected (*P* = .03), if 7 or more BG voxels were invaded at the time of diagnosis (*P* = .04), and/or the anterior portion of the AF, IFOF, anterior portion of IC (considered eloquent white matter) on the left side were invaded within the A3C2‐S2 voxel (*P* = .04; Figure 4).

In patients with oligodendrogliomas, a shorter OS was demonstrated if preoperative volume was larger than 62 mL (*P* = .02), and if the number of BG voxels was higher than 10 (*P* = .002).

The effect of EOR included more than two groups. With additional paired‐wise analysis of the surgical results, the patients with diffuse astrocytomas had a better OS if the EOR was >90% compared with an EOR of <70% (*P* = .02). In patients with oligodendrogliomas, a surgical resection rate >90% did not show an impact in OS compared with 70‐90% and <70% EOR (Figure 4).

In the logistic regression model, the univariate analysis of hazard ratio demonstrated that the side of invasion and the EOR were statistically significant predictors of OS (Table 2) in patients with diffuse astrocytomas. The number of Brain‐Grid voxels and the infiltrated eloquent white matter or the IDH status did not correlate with a significative impact on OS (Table 2).

**Table 2:** The upper part of the table shows the univariate OS analyses. Proportional hazards models were used to assess the prognostic significance of multiple variables OS in the two different groups: astrocytomas and oligodendrogliomas. The IDH status (IDH mutant, IDHm, or IDH wild type, IDHwt) was tested in astrocytomas only. The comparison between the molecular confirmation against the not otherwise specified (NOS) groups was tested in both astrocytomas and oligodendrogliomas

Analyzed variables	Astrocytomas			Oligodendrogliomas		
	*P*	HR	95% CI	*P*	HR	95% CI
Univariate analysis						
Side of invasion ‐ left/right/bilateral	.012*	0.108	0.019–0.611	.614	1.709	0.212‐13.748
Radiological border ‐ bulky/diffuse	.319	1.613	0.629‐4.137	.172	0.355	0.080‐1.570
Volume ‐ >O c‐off	.122	0.508	0.216‐1.197	.022*	0.314	0.117‐0.848
Brain‐Grid voxels ‐ >O c‐off	.062	0.433	0.180‐1.043	.017*	3.992	1.279‐12.455
Eloquent WM (BG) ‐ infiltrated	.063	2.327	0.956‐5.668	.904	1.064	0.386‐2933
Extent of resection ‐ GTR/STR	.044*	0.363	0.135‐972	.619	0.772	0.279‐2.141
IDH status ‐ IDHm/IDHwt	.522	1.238	0.644‐2.337			
Molecular status ‐ Confirmed/NOS	.756	1.171	0.432‐3.176	.902	1.078	0.324‐3.589
Multivariate analysis						
Side of invasion ‐ bilateral	.018*	2.997	1.204‐7.458			
Volume ‐ >O c‐off				.006*	1.012	1.003‐1.021
Interaction analysis						
Volume _×_ EOR	.780	0.995	0.964‐1.028	.943	1.002	0.950‐1.056
Volume _×_ Brain‐Grid voxels	.668	1.692	0.153‐18.719	.280	7.703	0.190‐312.645
Eloquent WM (BG) _×_ EOR	.629	0.994	0.972‐1.017	.915	0.999	0.981‐1.018
Brain‐Grid voxels _×_ EOR	.629	0.994	0.991‐1.031	.117	0.968	0.929‐1.008

Note

The middle part of the table shows the forward step‐wise proportional hazards modeling which was performed to assess the relative and independent prognostic capacity of each parameter. These two variables were selected by the equation as independent prognostic factors. The lower part of the table shows the interaction analysis as second block of the multivariate analysis for the most relevant variables: volume, EOR, Brain‐Grid voxels and eloquent white matter.

Abbreviations: HR, hazard risk; O c‐off, Optimal cutoff defined by ROC curves; OS, Overall survival.

* Statistically significant for *P < *.05.

In patients with oligodendrogliomas, preoperative large tumor volume (>62 mL) and the number of BG voxels (>10) demonstrated a statistically significant predictor for shorter OS (*P* < .05; Table 2).

Multivariate modeling was performed for each outcome measure using the following parameters: age, side of tumor, radiological border (bulky or diffuse), volume, involvement of eloquent white matter, Brain‐Grid voxels, EOR, and molecular verification status. In the multivariate model, bilateral tumor extension was the only independent predictor of shorter OS for patients with astrocytomas (*P* = .018, HR 2.997, 95% CI 1.204‐7.458). The volume, larger than 62 mL at diagnosis was the only independent predictor of shorter OS in the patients with oligodendrogliomas (*P* = .006, HR 1.012, 95% CI 1.003‐1.021). The second level analysis between the variables demonstrated no significant interaction as increased risk for a shorter OS neither in astrocytomas nor in oligodendrogliomas (Table 2).

## 4 DISCUSSION

Our results showed no significant difference between the molecularly confirmed subgroups (IDHm vs IDHwt) and between confirmed and NOS groups for any of the radiological or clinical features. Despite this result is in contrast with general biological implications regarding IDHwt diagnosis,^1^, ^27^, ^28^ we believe that they represent the very early stage of a more aggressive tumor with a similar radiological features.^28^ Hence, since our aim was to investigate radiological topographical features in diffuse gliomas, the tumors were divided in two main groups and compared based on their histological origin.

The most important result provided by our study is the clear difference in preferential location between histological types of diffuse gliomas. Astrocytomas are preferentially located fronto‐temporo‐insular on the left side, while oligodendrogliomas infiltrate the deep white matter of the frontal lobes bilaterally. Previous articles investigating preferential locations of diffuse gliomas, where different histological subtypes and grades were considered as one single entity, did not show similar distributions.^2^, ^3^, ^5^, ^19^, ^29^ Our results seem to radiologically confirm other studies suggesting a variability in terms of molecular biology among diffuse gliomas with regard to the tumor location,^8^, ^9^, ^10^ with a higher rate of 1p deletion in the anterior part of the brain (in particular in the frontal lobe)^9^ and a lower rate in the insula.^8^

The reasons for these differences are still unclear. Previous studies described LGGs as preferentially located in “secondary” functional areas, especially within the SMA and the insular lobe.^5^, ^6^, ^7^ Some authors suggest that preferential localization might be explained by developmental, cyto‐ and myeloarchitectectonic, neurochemical, metabolic, and functional reasons.^5^, ^8^ This could be possible especially regarding the insular localization, considering the anatomic, cytoarchitectonic, and functional interface between the allocortex (limbic system) and the neocortex.^5^, ^8^, ^30^ However, when we looked at the differences between the groups regarding white matter localization/infiltration, the role of cortices as the origin of these tumors became less prominent.

Diffuse astrocytomas tended to significantly invade association and projection white matter pathways between the insula and ventricles (Figures 2 and 3) within the fronto‐temporo‐insular region (A3C2S2). The anterior‐ventral portion of IFOF, the dorsal component of UF the anterior portion of AF, and the anterior ventral fibers system related to the internal capsule on the left side were the most frequently involved white matter pathways.

Diffuse oligodendrogliomas on the other hand, infiltrate larger and more heterogeneous white matter networks (association‐commissural‐projection white matter pathways), preferentially between basal ganglia and the deep and mesial regions of the frontal lobe bilaterally (Figures 2 and 3). The IFOF and frontal aslant tract (FAT) bilaterally, genu and body of corpus callosum, the anterior portion of the AF mostly on the right side, the anterior portion of anterior thalamic radiation (ATR) and internal capsule fibers on both sides were invaded with the highest frequency within the frontal mesial voxels bilaterally (A2‐3C2‐S1‐2; Figures 1, 2, 3).

These topographical differences in the regions involved bear some similarities with the mechanisms of cell migration during brain development.^31^, ^32^, ^33^ The insula is among the first cortical regions to appear, and its development is linked to radial glial migration, which establishes the general cyto‐architectonical framework of the different forebrain subdivisions from the ventricular zone to pia.^31^, ^33^, ^34^ After neuronal production ceases, radial glial cells retract their ventricular and pial attachments and differentiate into astrocytes.^31^, ^35^, ^36^, ^37^ The process of oligodendrocytes migration is regulated by a more prominent tangential migration, which increases the cellular complexity of forebrain circuits following preferential white matter tracts from the globus pallidus internus (in human, anterior entopeduncular nucleus in rodents and birds) to anterior and dorsal cortical regions.^31^, ^32^, ^38^, ^39^, ^40^

The differences in preferential location and white matter pathways invaded by tumors may reflect the different biological behaviors of the two groups of glia cells, suggesting abnormalities in cell migration at some point in the developmental or the adult stage.^31^, ^33^

Differences in types and amount of invaded white matter pathways may also reflect differences in terms of prognosis. Diffuse astrocytomas that preferentially invaded association and projection white matter networks on the left side within the fronto‐temporo‐insular BG voxel (A3C2S2) have a significantly reduced OS (*P* = .02; Figure 4). If the eloquent white matter pathways were infiltrated, the patients had a slightly higher risk of shorter OS (HR 2.32), but this was, however, not statistically significant (*P* = .06; Table 2). The link between the impact of eloquent white matter and neurological deficits in LGGs has been described in other studies that have shown surgical implications and limitations to speech and cognitive functions when the so‐called “minimal common brain” on the left side was invaded.^19^, ^41^, ^42^, ^43^ However, in our study only patients with diffuse astrocytomas displayed this impact of white matter on OS.

One possible reason may be the impact of surgical resection when the eloquent white matter is infiltrated. The importance of EOR for the outcome in LGG patients is widely accepted.^44^, ^45^ A supratotal resection or gross total resection is recommended to effectively change the natural history of this disease and possibly postpone anaplastic transformation.^42^, ^46^, ^47^ This achievement may be more difficult in astrocytomas than in oligodendrogliomas, where localization (closer to central core/invading basal ganglia) does not often allow a radical resection.^19^, ^48^, ^49^ Considering the locations and the white matter structures involved, the use of intraoperative monitoring is nowadays mandatory to preserve high order functions and an oncological‐functional balance in low‐grade gliomas.^3^, ^45^, ^49^ The advantages of awake surgery represents the best option when surgical management for these lesions is possible.^19^, ^43^ This retrospective study has been performed at the beginning of our learning curve on the modern management of low‐grade gliomas, and therefore awake surgery was not offered to any of these patients. Despite this limitation, surgical treatment of diffuse astrocytomas that achieved an EOR > 90% was a prognostic factor for a longer OS compared to other surgical strategies (Table 2; Figure 4). On the other hand, bilateral infiltration represented an independent factor for shorter OS in the same group (Table 2). This may be explained by a difference in the surgical management of these lesions. Tumors that had widely invaded neighboring structures were often managed with diagnostic biopsy instead of more aggressive strategy.^42^, ^48^, ^49^

In our study, the two groups also differed in their grade of invasiveness. We merged information on radiological borders, tumor volume (median volume of 46 mL for astrocytomas vs 62 mL for oligodendrogliomas), and number of Brain‐Grid voxels (median number of 7 for astrocytomas, 8 for oligodendrogliomas) at the radiological diagnosis to better detect the invasive behavior of each group. Radiological borders did not differ between the two groups (or subgroups) even if oligodendrogliomas displayed a higher rate of diffuse tumor (72%). Despite its potential importance, this aspect was, however, not correlated with a different OS (Table 2 and Figure 4). Tumor volume computation, on the other hand, represents a gold standard technique for LGG observation and prognosis.^29^, ^45^, ^50^, ^51^, ^52^ In our study, oligodendrogliomas showed a larger volume at diagnosis (62 vs 46 mL) but without significative difference between the two groups (Table 1). Analyzed by their impact on OS, only oligodendrogliomas displayed a strong effect of preoperative volume on a shorter OS (Table 2; Figure 4). We believe that this result should be carefully analyzed in the light of the intrinsic limitation of the 3‐D volume computation: the lack of related topographical and qualitative information.^16^ Tumors with the same volume can behave differently based on their location and radiological borders (bulky or diffuse). When only these two parameters are considered, we were unable to detect tumor invasiveness. The BG system allowed us to identify a new quantitative difference between the two groups in terms of invasiveness. The critical number of 7 BG voxels for astrocytomas and 10 BG voxels infiltrated for oligodendrogliomas, significantly predicted the probability of a shorter OS in both groups (*P* < .05; Figure 4) and more consistently in oligodendrogliomas, where it can be considered a risk factor for the prognosis (Table 2). The number of BG voxels can represent a new prognostic variable able to predict tumor invasiveness and its impact on OS and thereby suggest an advanced stage of tumor infiltration at the time of radiological diagnosis. Therefore, we suggest that this system should be used as complementary observational tool together with 3D tumor volume computation for a better pre‐surgical planning and, for a better observation of tumor kinetics.

In summary, astrocytomas and oligodendrogliomas were very different in terms of anatomical/topographical features and invasiveness at the time of radiological diagnosis.

Diffuse astrocytomas displayed a preferential location for fronto‐temporo‐insular region on the left side and a smaller average volume, often with a diffuse radiological border. They tended to infiltrate association and projection white matter pathways between the insula and ventricles, and the critical cutoff of 7 BG voxels reflected a higher invasiveness able to predict a shorter OS.

Diffuse oligodendrogliomas, on the other hand, showed a preferential location for the subcortical region of the frontal lobes bilaterally, with larger volume and very often diffuse radiological borders. They preferentially infiltrated association‐commissural‐projection white matter pathways between basal ganglia and the deep and mesial regions of the frontal lobe bilaterally, and the critical number of 10 BG voxels indicated a high tumor invasiveness that was able to significantly predict a shorter OS.

We believe that a more extensive comprehension of the differences among subtypes of diffuse gliomas is essential to achieve better results with regard to OS. The information provided in this study can contribute to a better and more detailed classification of diffuse gliomas through identification of differences in preferential location, a better prediction of biological behavior, and improved presurgical planning.

### 4.1 Limitations

This study has some limitations. First, the histological diagnosis of the included patients may be incomplete according to the WHO‐2016 classification criteria for brain tumors. The IDH mutation analysis and the 1p19q codeletion are the gold standard nowadays for the diagnosis and classification of gliomas.^1^ Because of the retrospective nature of this study, not all the patients had a molecular analysis available at the time of treatment and the methods and the histo‐immunological criteria changed dramatically in the last 15 years. However, all the previously mixed oligoastrocytomas were reviewed in light of the new classification criteria. Moreover, the proliferation index was taken into consideration and the histologically WHO‐III gliomas were not included in this case series showing our effort to analyze a more homogeneous population. Hence, all the NOS patients were included into the respective subgroups of histological diagnoses. Although this brings some diagnostic faults into the main groups, as the subgroup of tumors without the molecular classifications (LOH 1p19q and IDH, ie, NOS) will somewhat obscure the results, the differences we do see in the study, would most likely be larger rather than those presented, had all the tumors been molecularly classified. In addition, since no differences were detected in clinical or radiological variables among the subgroups (not even between IDHm and IDHwt), the topographical analysis was performed through gradient/probabilistic maps on the two main groups (astrocytomas and oligodendrogliomas). Despite the already discussed differences in molecular profiles, we believe that this was the best choice to detect differences/similarities in tumor topography based on their histological origin.

A second criticism may arise regarding the intrinsic features of the chosen variables. One may observe as expected that some variables, that is, volume and BG voxels, as well as infiltration of crucial white matter and resection grade may be related to each other. We choose to not perform a correlation analysis which was beyond our aim. Correlation is used to determine the direction and strength of the relationship between two variables which in our case are well known to move together (ie, the effect of eloquent white matter on surgical resection, or the volume and number of BG voxels). Our aim was not to investigate the impact of a treatment or to find a numerical value expressing the relationship between the variables. With the regression model we wanted to determine the effect of independent variables on the OS and try to determine its variation.

The interaction analysis between the more relevant variables did not show a significantly combined effect on the HR supporting our decision. Aware of these limitations we believe that our results show substantial differences among topographical behavior of two different pathologies that share some molecular characteristics. Further biological studies, rather prospective, and with larger cohorts of patients will be able to confirm these results.

## 5 CONCLUSION

Diffuse astrocytomas and oligodendrogliomas display differences in preferential topographical location. This difference reflects the type and the amount of white matter structures involved at the time of radiologic diagnosis and affects the whole course of this disease. Tumor invasiveness analyzed with the BG system predicted shorter OS in both groups of tumors. This new information may be valuable in neurosurgical oncology to classify and plan the best treatment strategy in patients with diffuse gliomas.

## CONFLICT OF INTEREST

All authors certify that they have no affiliations with or involvement in any organization or entity with any financial interest (such as honoraria; educational grants; participation in speakers' bureaus; membership, employment, consultancies, stock ownership, or other equity interest; and expert testimony or patent‐licensing arrangements), or nonfinancial interest (such as personal or professional relationships, affiliations, knowledge, or beliefs) in the subject matter or materials discussed in this manuscript.

## AUTHOR CONTRIBUTION

Contributed to the experimental design and its implementation: FL, MF, MR; Analysis and interpretation of the data: FL, MF; Writing of the manuscript at draft and any revision stages: all the authors; Read and approved the final version: all the authors.

## Data Availability

The data that support the findings of this study are available on request from the corresponding author. The data are not publicly available due to privacy or ethical restrictions.

## References

[1] Louis DN , Perry A , Reifenberger G , et al. The 2016 World Health Organization classification of tumors of the central nervous system: a summary. Acta Neuropathol. 2016;131:803‐820. 27157931 10.1007/s00401-016-1545-1

[2] Corell A , Carstam L , Smits A , Henriksson R , Jakola AS . Age and surgical outcome of low‐grade glioma in Sweden. Acta Neurol Scand. 2018;138:359‐368. 29900547 10.1111/ane.12973

[3] Schiff D . Low‐grade gliomas. Continuum (Minneap Minn) 2017;23:1564‐1579. 29200111 10.1212/CON.0000000000000537

[4] Ohgaki H , Kleihues P . The definition of primary and secondary glioblastoma. Clin Cancer Res. 2013;19:764‐772. 23209033 10.1158/1078-0432.CCR-12-3002

[5] Duffau H , Capelle L . Preferential brain locations of low‐grade gliomas. Cancer. 2004;100:2622‐2626. 15197805 10.1002/cncr.20297

[6] Larjavaara S , Mäntylä R , Salminen T , et al. Incidence of gliomas by anatomic location. Neuro Oncol. 2007;9:319‐325. 17522333 10.1215/15228517-2007-016 PMC1907421

[7] Parisot S , Darlix A , Baumann C , et al. A Probabilistic atlas of diffuse WHO grade II glioma locations in the brain. PLoS ONE 11: 2016 https://www.ncbi.nlm.nih.gov/pmc/articles/PMC4709135/. Accessed March 31, 2019 10.1371/journal.pone.0144200 PMC4709135 26751577

[8] Gozé C , Rigau V , Gibert L , Maudelonde T , Duffau H . Lack of complete 1p19q deletion in a consecutive series of 12 WHO grade II gliomas involving the insula: a marker of worse prognosis? J Neurooncol. 2009;91:1‐5. 18726074 10.1007/s11060-008-9680-8

[9] Laigle‐Donadey F , Martin‐Duverneuil N , Lejeune J , et al. Correlations between molecular profile and radiologic pattern in oligodendroglial tumors. Neurology. 2004;63:2360‐2362. 15623700 10.1212/01.wnl.0000148642.26985.68

[10] Metellus P , Coulibaly B , Colin C , et al. Absence of IDH mutation identifies a novel radiologic and molecular subtype of WHO grade II gliomas with dismal prognosis. Acta Neuropathol. 2010;120:719‐729. 21080178 10.1007/s00401-010-0777-8

[11] Stockhammer F , Misch M , Helms H‐J , et al. IDH1/2 mutations in WHO grade II astrocytomas associated with localization and seizure as the initial symptom. Seizure. 2012;21:194‐197. 22217666 10.1016/j.seizure.2011.12.007

[12] Giese A , Westphal M . Glioma invasion in the central nervous system. Neurosurgery. 1996;39:235‐252; discussion 250–252, 1996. 8832660 10.1097/00006123-199608000-00001

[13] Schwartz MS , Morris J , Sarid J . Overexpression of oncogene products can cause tumor progression without parenchymal infiltration in the rat brain. Cancer Res. 1991;51:3595‐3601. 1675934

[14] Harsh GR IV , Wilson CB . Neuroepithelial tumors of the adult brain In: Youmans JR, ed. Neurological Surgery, 3rd edn Philadelphia: W.B. Saunders Co; 1990, vol 5: pp. 3040‐3136.

[15] Guthrie BL , Laws ER . Supratentorial low‐grade gliomas. Neurosurg Clin N Am. 1990;1:37‐48. 2135972

[16] Latini F , Fahlström M , Berntsson SG , Larsson E‐M , Smits A , Ryttlefors M . A novel radiological classification system for cerebral gliomas: the Brain‐Grid. PLoS ONE. 2019;14:e0211243. 30677090 10.1371/journal.pone.0211243 PMC6345500

[17] Latini F , Larsson E‐M , Ryttlefors M . Rapid and accurate MRI segmentation of peritumoral brain edema in meningiomas. Clin Neuroradiol. 2017;27:145‐152. 26603998 10.1007/s00062-015-0481-0

[18] Evans AC , Marrett S , Neelin P , et al. Anatomical mapping of functional activation in stereotactic coordinate space. NeuroImage. 1992;1:43‐53. 9343556 10.1016/1053-8119(92)90006-9

[19] Ius T , Angelini E , Thiebaut de Schotten M , Mandonnet E , Duffau H . Evidence for potentials and limitations of brain plasticity using an atlas of functional resectability of WHO grade II gliomas: towards a “minimal common brain”. NeuroImage. 2011;56:992‐1000. 21414413 10.1016/j.neuroimage.2011.03.022

[20] Van Essen DC , Smith SM , Barch DM , et al. The WU‐Minn Human Connectome Project: an overview. NeuroImage. 2013;80:62‐79. 23684880 10.1016/j.neuroimage.2013.05.041 PMC3724347

[21] Yeh F‐C , Tseng W‐YI . NTU‐ 90: a high angular resolution brain atlas constructed by q‐space diffeomorphic reconstruction. NeuroImage. 2011;58:91‐99. 21704171 10.1016/j.neuroimage.2011.06.021

[22] Yeh F‐C , Wedeen VJ , Tseng W‐YI . Generalized q‐sampling imaging. IEEE Trans Med Imaging. 2010;29:1626‐1635. 20304721 10.1109/TMI.2010.2045126

[23] Yeh F‐C , Liu L , Hitchens TK , Wu YL . Mapping immune cell infiltration using restricted diffusion MRI. Magn Reson Med. 2017;77:603‐612. 26843524 10.1002/mrm.26143 PMC8052951

[24] Kaplan EL , Meier P . Nonparametric estimation from incomplete observations. J Am Stat Assoc. 1958;53:457‐481.

[25] Carter JV , Pan J , Rai SN , Galandiuk S . ROC‐ing along: evaluation and interpretation of receiver operating characteristic curves. Surgery. 2016;159:1638‐1645. 26962006 10.1016/j.surg.2015.12.029

[26] Cox DR . Regression models and life‐tables. J Roy Stat Soc: Ser B (Methodol). 1972;34:187‐202.

[27] Hasselblatt M , Jaber M , Reuss D , et al. Diffuse astrocytoma, IDH‐wildtype: a dissolving diagnosis. J Neuropathol Exp Neurol. 2018;77:422‐425. 29444314 10.1093/jnen/nly012

[28] Reuss DE , Kratz A , Sahm F , et al. Adult IDH wild type astrocytomas biologically and clinically resolve into other tumor entities. Acta Neuropathol. 2015;130:407‐417. 26087904 10.1007/s00401-015-1454-8

[29] Jung T‐Y , Jung S , Moon J‐H , Kim I‐Y , Moon K‐S , Jang W‐Y . Early prognostic factors related to progression and malignant transformation of low‐grade gliomas. Clin Neurol Neurosurg. 2011;113:752‐757. 21889256 10.1016/j.clineuro.2011.08.002

[30] Augustine JR . Circuitry and functional aspects of the insular lobe in primates including humans. Brain Res Brain Res Rev. 1996;22:229‐244. 8957561 10.1016/s0165-0173(96)00011-2

[31] Marín O , Rubenstein JLR . Cell migration in the forebrain. Annu Rev Neurosci. 2003;26:441‐483. 12626695 10.1146/annurev.neuro.26.041002.131058

[32] Olivier C , Cobos I , Villegas EMP , et al. Monofocal origin of telencephalic oligodendrocytes in the anterior entopeduncular area of the chick embryo. Development. 2001;128:1757‐1769. 11311157 10.1242/dev.128.10.1757

[33] Zhan JS , Gao K , Chai RC , et al. Astrocytes in migration. Neurochem Res. 2017;42:272‐282. 27837318 10.1007/s11064-016-2089-4

[34] González‐Arnay E , González‐Gómez M , Meyer G . A radial glia fascicle leads principal neurons from the pallial‐subpallial boundary into the developing human insula. Front Neuroanat. 11: 2017 https://www.frontiersin.org/articles/10.3389/fnana.2017.00111/full. Accessed July 18, 2019 10.3389/fnana.2017.00111 PMC5723317 29259547

[35] Ge W‐P , Jia J‐M . Local production of astrocytes in the cerebral cortex. Neuroscience. 2016;323:3‐9. 26343293 10.1016/j.neuroscience.2015.08.057 PMC4779064

[36] Heins N , Malatesta P , Cecconi F , et al. Glial cells generate neurons: the role of the transcription factor Pax6. Nat Neurosci. 2002;5:308‐315. 11896398 10.1038/nn828

[37] Malatesta P , Hartfuss E , Götz M . Isolation of radial glial cells by fluorescent‐activated cell sorting reveals a neuronal lineage. Development. 2000;127:5253‐5263. 11076748 10.1242/dev.127.24.5253

[38] Ono K , Yasui Y , Rutishauser U , Miller RH . Focal ventricular origin and migration of oligodendrocyte precursors into the chick optic nerve. Neuron. 1997;19:283‐292. 9292719 10.1016/s0896-6273(00)80939-3

[39] Spassky N , Goujet‐Zalc C , Parmantier E , et al. Multiple restricted origin of oligodendrocytes. J Neurosci. 1998;18:8331‐8343. 9763477 10.1523/JNEUROSCI.18-20-08331.1998 PMC6792828

[40] Tripathi RB , Clarke LE , Burzomato V , et al. Dorsally‐ and ventrally‐derived oligodendrocytes have similar electrical properties but myelinate preferred tracts. J Neurosci. 2011;31:6809‐6819. 21543611 10.1523/JNEUROSCI.6474-10.2011 PMC4227601

[41] Duffau H . Diffuse low‐grade glioma, oncological outcome and quality of life: a surgical perspective. Curr Opin Oncol. 2018;30:383‐389. 30124519 10.1097/CCO.0000000000000483

[42] Jakola AS , Unsgård G , Myrmel KS , et al. Surgical strategies in low‐grade gliomas and implications for long‐term quality of life. J Clin Neurosci. 2014;21:1304‐1309. 24798909 10.1016/j.jocn.2013.11.027

[43] Sarubbo S , De Benedictis A , Merler S , et al. Towards a functional atlas of human white matter. Hum Brain Mapp. 2015;36:3117‐3136. 25959791 10.1002/hbm.22832 PMC6869563

[44] Hervey‐Jumper SL , Berger MS . Evidence for improving outcome through extent of resection. Neurosurg Clin N Am. 2019;30:85‐93. 30470408 10.1016/j.nec.2018.08.005

[45] Smith JS , Chang EF , Lamborn KR , et al. Role of extent of resection in the long‐term outcome of low‐grade hemispheric gliomas. J Clin Oncol. 2008;26:1338‐1345. 18323558 10.1200/JCO.2007.13.9337

[46] Duffau H . The role of surgery in low‐grade gliomas: do timing and extent of resection matter? CNS Oncol. 2017;6:179‐183. 28718304 10.2217/cns-2017-0009 PMC6011168

[47] McKhann GM , Duffau H . Low‐grade glioma: epidemiology, pathophysiology, clinical features, and treatment. Neurosurg Clin N Am. 2019:30;xiii‐xiv. 10.1016/j.nec.2018.10.001 30470410

[48] Aghi MK , Nahed BV , Sloan AE , Ryken TC , Kalkanis SN , Olson JJ . The role of surgery in the management of patients with diffuse low grade glioma: a systematic review and evidence‐based clinical practice guideline. J Neurooncol. 2015;125:503‐530. 26530265 10.1007/s11060-015-1867-1

[49] Hottinger AF , Hegi ME , Baumert BG . Current management of low‐grade gliomas. Curr Opin Neurol. 2016;29:782‐788. 27676279 10.1097/WCO.0000000000000390

[50] Freyschlag CF , Krieg SM , Kerschbaumer J , et al. Imaging practice in low‐grade gliomas among European specialized centers and proposal for a minimum core of imaging. J Neurooncol. 2018;139:699‐711. 29992433 10.1007/s11060-018-2916-3 PMC6132968

[51] Pallud J , Blonski M , Mandonnet E , et al. Velocity of tumor spontaneous expansion predicts long‐term outcomes for diffuse low‐grade gliomas. Neuro‐Oncology. 2013;15:595‐606. 23393207 10.1093/neuonc/nos331 PMC3635513

[52] Still MEH , Roux A , Huberfeld G , et al. Extent of resection and residual tumor thresholds for postoperative total seizure freedom in epileptic adult patients harboring a supratentorial diffuse low‐grade glioma. Neurosurgery. 2019;85:E332‐E340. 30395304 10.1093/neuros/nyy481

